# The pharmacological treatment of schizophrenia: How far have we come?

**DOI:** 10.1002/pcn5.13

**Published:** 2022-05-30

**Authors:** Jose M. Rubio, John M. Kane

**Affiliations:** ^1^ Donald and Barbara Zucker School of Medicine at Hofstra—Northwell, Feinstein Institutes of Medical Research—Institute of Behavioral Science Zucker Hillside Hospital—Northwell Health Glen Oaks NY United States

**Keywords:** antipsychotic drugs, coordinated specialty care, recovery‐oriented care

## Abstract

Schizophrenia is a chronic and often severe mental disorder for which antipsychotic drugs are the cornerstone of treatment. Although the essential mechanism of action of these drugs has not changed much since they were first discovered in the 1950s, there have been numerous advances in the context in which these drugs are prescribed, as well as in the considerations for their optimal use. In this review, we summarize five selected issues in which the psychopharmacological treatment of schizophrenia has most evolved. Namely, these are the shift of outcomes of interest from symptoms to recovery, the development of stratified approaches to select the most appropriate treatment for each individual, the recognition of treatment nonadherence as a critical factor determining outcomes, the recommendations for maintenance treatment, and, finally, the promise of new antipsychotic compounds that innovate in their mechanisms of action, improving efficacy/safety profiles. Finally, we discuss how some of these advances have already delivered to improved outcomes in the real world, whereas others have demonstrated efficacy under optimal circumstances yet have not been translated into better outcomes in the community. Thus, the road ahead includes both identifying novel treatments that engage the psychopathology of the illness and improve the efficacy/tolerability profile of currently available agents, as well as developing interventions that mitigate the barriers for the use of novel interventions, some of them already existing, in the real world.

## INTRODUCTION

Schizophrenia is a chronic disorder characterized by positive symptoms (i.e., hallucinations, delusions), negative symptoms (i.e., affective flattening, amotivation), and cognitive symptoms (working memory, abstraction).^1^ These experiences have tremendous impact on social and psychological functioning as do common medical comorbidities. Given that schizophrenia typically begins at an early age, the result can be long‐term disability and premature mortality.^2^, ^3^ Antipsychotic drugs are the cornerstone of the treatment of schizophrenia. These drugs were initially developed in the 1950s, in what became the beginning of the modern era in the pharmacological treatment of schizophrenia.^4^ In this article we aim to summarize the progress made over recent years in selected areas in the psychopharmacology of schizophrenia, namely recovery‐oriented care, precision medicine, management of treatment nonadherence, relapse prevention, and novel mechanisms of action of antipsychotic drugs. We highlight the most relevant examples for each one of these areas in Table 1.

**Table 1:** Key developments in the treatment of schizophrenia

**Pharmacology in the context of recovery‐oriented care**
‐ Symptom control is necessary, but not sufficient for recovery, which is increasingly being recognized as an outcome of interest
‐ Psychopharmacology interventions should be embedded within a comprehensive approach that addresses all symptom domains in schizophrenia, as well as social and psychological outcomes and medical and psychiatric comorbidities, in alignment with each individual's recovery goals
‐ Treatment teams should balance between shared decision‐making and motivational interviewing to address these goals
**Precision medicine approaches**
‐ Stratification approaches for the treatment of schizophrenia do exist for treatment resistance (i.e., clozapine after two failed trials), but this remains an underutilized resource
‐ Prognostic biomarkers of treatment response for personalized medicine are being developed and show promise, in some cases with validation of results in independent samples
**Addressing the challenges of treatment adherence**
‐ Nonadherence is prevalent and challenging to detect in a reliable and scalable manner
‐ Long‐acting injectable antipsychotics are the most effective approach to reduce the impact of nonadherence on treatment
**Evidence‐based maintenance treatment recommendations**
‐ Maintenance treatment with antipsychotics is highly effective in preventing relapses
‐ Current guidance recommends using full doses for stabilization
‐ There is no direct data to recommend duration of treatment beyond ~2 years after stabilization, but indirect evidence suggests that most individuals with schizophrenia may need to continue treatment over the long term
**Novel mechanisms of action**
‐ 60 years after the development of antipsychotic medicines, there are promising developments in drugs with alternative mechanisms of action to dopamine 2 receptor interaction
‐ TAAR‐1 agonists still interact with the dopaminergic system, but not through the dopamine 2 receptor, and have demonstrated promising results in phase 3 studies
‐ Similarly, xanomeline‐trospium, which interacts with the cholinergic system, has shown promising efficacy data in phase 3 studies

## FROM SYMPTOM‐FOCUSED TREATMENT TO PSYCHOPHARMACOLOGY IN THE CONTEXT OF RECOVERY‐ORIENTED CARE

The outcomes of interest in the treatment of schizophrenia have evolved over the last several decades, from a focus on symptom severity, to the broader impact of schizophrenia on various life domains. Thus, in addition to positive, negative, and cognitive symptoms, there is increasing attention on the social, vocational, and psychological consequences of the illness, such as limited social support, unemployment, homelessness, or stigma, as intervention targets.^5^ Despite the growing attention to recovery in schizophrenia, the data on how often this outcome is achieved are still disappointing. In a 2013 meta‐analysis,^6^ recovery in schizophrenia, understood as at least 2 years of mild symptoms plus social functioning outcome greater to an equivalent of 61 in the global assessment of function (GAF), occurred in 13.5% of the meta‐analyzed studies. Furthermore, there was no evidence that recovery rates had improved over the preceding five decades. Early intervention services (EIS), which contextualize psychopharmacological treatment within the need to address the social and psychological aspects of the illness, have emerged in response to such low recovery rates in an attempt to improve psychopathology as well as functional outcomes. The focus of these programs is on the early phase of the illness as a critical time for recovery‐oriented interventions, since individuals generally respond better to treatment and they have not yet endured years of functional decline.^7^ In addition, it has been suggested that better control over the illness in the first 2 years is an important prognostic indicator.^8^ EIS constitutes at minimum regularly monitored psychopharmacological treatment, family psychoeducation, and counseling. However, very often they may also include cognitive behavior therapy for psychosis, family therapy, supportive education and employment, social skills training, and crisis management.^7^

Recovery‐oriented care in general, and in particular EIS for the treatment of schizophrenia, is progressively permeating the mental healthcare system in the United States. In 2015, the United States Congress began setting aside funds to support the implementation of coordinated specialty care (CSC), a specific package of EIS, across the country.^9^ This healthcare policy decision was supported by the results of the Recovery After an Initial Schizophrenia Episode (RAISE) study, funded by the National Institute of Mental Health.^10^ In a cluster randomized clinical trial, the RAISE‐early treatment program (ETP) study compared CSC to treatment as usual (TAU) in 34 nonacademic clinics across the United States with real‐world funding contingencies. For this study, CSC was formulated as a recovery‐oriented treatment program that promotes shared decision‐making and uses a single point of access for coordinated psychotherapy, family education and support, case management, supportive education and employment, and specialized medication management. Individuals receiving treatment in clinics randomized to CSC remained in treatment longer, experienced greater improvement in quality of life and psychopathology, and experienced greater involvement in school or work than those who received usual care.^11^ Such gains were particularly prominent among those who had experienced shorter duration of untreated psychosis, highlighting the relevance for early intervention. In fact, EIS at large, not just CSC, have demonstrated superior effectiveness in the treatment of schizophrenia. In a 2018 meta‐analysis of 10 studies conducted in several different countries^12^ comparing EIS to TAU, EIS were significantly superior to TAU in all 13 meta‐analyzed outcomes, ranging from treatment discontinuation to involvement in school or work to severity of psychopathology, with small to moderate effect sizes.

In this context, the pharmacological treatment of schizophrenia focuses on the following: (1) using of evidenced‐based practices and measurement‐based care to inform the shared decision‐making process; (2) aiming for symptom remission rather than only response; (3) using antipsychotics with favorable side effect profiles; (4) using low doses; (5) monitoring side effects closely; (6) monitoring medical and substance use comorbidities; (7) using clozapine after two failed antipsychotic trials; and (8) normalizing nonadherence and intervening accordingly.^13^ Such recommendations are based on a compelling amount of literature, some of which we will expand on later in this review. Nevertheless, despite this body of literature, there is still work to do to translate these best practices into real‐world outcomes. In the RAISE‐ETP study, ~40% of individuals had a psychopharmacology regimen that deviated from these basic recommendations,^14^ and a cohort of 6246 individuals from a Medicaid claims data set found that a similar proportion of patients with early phase schizophrenia were receiving treatment that deviated from these principles.^15^ This finding is not completely surprising given the known statistic that evidence‐based recommendations may take close to two decades to transition from publication to adoption in routine care.^16^

Additional outcomes of interest for the care of those living with schizophrenia, beyond psychopathology and functional outcomes, are those related to substance use and medical comorbidities. Almost half of the individuals with schizophrenia will also suffer from a substance use disorder at some point in their lives.^17^ Importantly, this comorbidity is linked to poorer clinical outcomes in the domains of psychopathology,^18^ functional outcomes,^19^ and physical health.^20^ For example, substance use comorbidity increases by more than twofold the risk of schizophrenia relapse,^21^ and is associated with greater rates of persistent depression and poorer quality of life over time,^22^ as well as greater rates of poorer treatment compliance, violence, incarceration, housing instability, and homelessness.^23^ The reason for the strong association between these two types of disorders is complex. The mesolimbic dopamine pathway has been involved in the reward mechanism for drugs^24^ as well as in the aberrant salience associated with positive symptoms in schizophrenia.^25^ Thus, it is conceivable that the two share genetic liability that manifests in maladaptive reward processing,^26^ and/or one predisposes to the other (e.g., substance use to compensate for deficits resulting from aberrant signaling in the mesolimbic dopamine pathway).^27^ One of the comorbidities with most implications, because of its impact and prevalence, is nicotine smoking. Although overall the prevalence of smoking is decreasing in many countries,^28^ this still represents an important problem for those living with schizophrenia. In the RAISE study, despite the relatively young age of study participants, the prevalence of smoking was about 50%,^29^ compared to about 14% in the general population.^30^ Such high rates of smoking have been associated with lung cancer, chronic obstructive pulmonary disease, and cardiovascular disease, making nicotine smoking one of the most powerful drivers of premature mortality among those living with schizophrenia.^31^

In fact, physical health and premature mortality have gained attention in recent years, given the worrisome statistic that individuals with schizophrenia live an average of about 15 years less than those without schizophrenia.^32^ In addition to nicotine smoking, other modifiable factors are also involved. There are consistent data from Scandinavian registries reporting shorter life expectancy for those with lower cumulative doses of antipsychotic treatment,^33^ suggesting that long‐term adherence with relapse‐prevention treatment may result in longer life expectancy. Other modifiable risk factors include obesity and metabolic syndrome. The relationship with metabolic disturbance, mortality, and antipsychotic drugs is complex, since although antipsychotics are known to cause metabolic disturbance,^34^ this is very likely offset in terms of risk of premature mortality by better symptom control and higher levels of functioning, which would facilitate primary prevention and better management of medical comorbidities in this population. Clients and prescribers should be careful about monitoring the impact of antipsychotic side effects by screening for metabolic effects regularly,^35^ managing or referring to primary care if disorders of metabolism are identified, and choosing antipsychotic agents carefully. In addition, it is important to treat co‐occurring substance use, address sedentary lifestyles, and involve case management when possible and/or appropriate to facilitate coordinating care among psychiatric and medical providers.^36^

What is the role of the prescriber in the framework of recovery‐oriented care, given the intervention targets of symptoms, functioning, substance use comorbidities, and physical health? In addition to an expansion of the outcomes of interest, recovery‐oriented care emphasizes the active role of the client in treatment choice.^5^ Thus, the role of the prescriber, in what is known as shared decision‐making,^37^ is to identify the patient preferences and values in regard to control of psychopathology, goals of psychosocial and vocational functioning, and physical health in order to support the patient's choice among various treatment options, each of them with alignments and misalignments with the patient's goals. It is not infrequent to observe ambivalence in the goals of care, or misalignments between the goals and the known consequences of the healthcare choices. An example is someone who wants to be able to go back to work after an acute psychotic episode, but who chooses to discontinue antipsychotic maintenance treatment after having relapsed in the past on previous treatment interruptions. This scenario may call for motivational interviewing,^38^ in which the role of the prescriber is not to only provide information about treatment options, but also to help the client solve the ambivalence between goals and choices by formulating questions whose answers may point towards those contradictions.

## FROM ONE SIZE FITS ALL TO PRECISION MEDICINE APPROACHES

Precision medicine consists of the exercise of clinical decisions in response to the individual's specific biological and environmental make‐up.^39^ This seems necessary considering the heterogeneity of clinical outcomes to treatment interventions. The prognosis of schizophrenia ranges from a small minority of individuals for whom there will never be a second episode of acute psychosis, to another proportion that will experience persistent and debilitating symptoms despite exhausting all treatment options.^1^ In addition, antipsychotic drugs, which are the main treatment in schizophrenia, often are associated with side effects such as weight gain, metabolic disturbance, or tardive dyskinesia,^35^, ^40^ which makes it necessary to be able to inform treatment decisions so that efficacy and effectiveness are maximized while the price paid in terms of side effects is minimized. Other areas of medicine, like oncology or cardiology, have incorporated into their clinical decision process input about the genetic profile of the individual in order to recommend a specific treatment.^41^ In the treatment of schizophrenia, such stratification of treatment is conducted primarily based on clinical markers, although there is promising research indicating that biomarkers may inform those treatment decisions in the future. The first of such clinical markers was history of failure to respond to two trials of sufficient dose and time with antipsychotic drugs.^42^ There is abundant data indicating that individuals who will fail to respond to nonclozapine antipsychotics may be more likely to respond to clozapine,^43^ suggesting possibly a distinct neurobiology^44^ and certainly the need to stratify treatment accordingly. The criteria to define so‐called “treatment resistance,” and thus of clozapine eligibility, has been recently agreed on,^45^ and in essence it applies to subjects for whom the trials were at least 6 weeks in duration, with an equivalent daily dose of at least 600 mg of chlorpromazine, for which adherence could be demonstrated (either by a therapeutic plasma level of antipsychotic medicine or by a long‐acting injectable [LAI] trial), and for whom there was residual psychotic symptoms as well as functional impairment. Recent research has advocated to modify such a clinical marker for clozapine eligibility to one failed trial.^46^ However, this remains controversial given the limitations of the data used to make such a recommendation, including lack of a control condition or the possibility of treatment nonadherence with only one trial, which is particularly high in the earlier phases of treatment.^47^ Despite the compelling evidence and clear criteria to stratify treatment according to previous treatment history, especially after two failed trials, the use of clozapine is often delayed for years^48^ and overall usage is much lower than would be expected based on the incidence of treatment‐resistant schizophrenia.^49^

Another clinical marker that can be used to inform clinical decisions is early nonresponse (ENR). Failure to minimally respond to antipsychotic treatment during the initial weeks of treatment has been confirmed as a valid prognostic paradigm of treatment response. For instance, in a recent meta‐analysis of 43 studies, Samara et al.^50^ examined the prognostic capability of early nonresponse (ENR) to predict failure to a trial of antipsychotic drugs. The authors found that improvements in psychotic symptom severity <20% after 2 weeks of treatment had a specificity of 86% and a negative predictive value of 90% in predicting poor treatment response. The authors found that the predictive ability of ENR was consistent across study populations and characteristics. Such low likelihood of treatment response in patients who do not even minimally improve during the initial weeks of treatment has resulted in recommendations to expedite treatment change for these individuals in recently updated guidelines.^51^

Unfortunately, one limitation of using clinical markers to inform subsequent treatment decisions is that they rely on trial and error, which means longer time experiencing symptoms until the optimal treatment is identified. One promise of prognostic biomarkers is that they can leverage objective measurements related to the pathophysiology of the illness to inform treatment decisions before engaging in trial and error. In schizophrenia, the converging evidence for aberrant striatal function in the pathophysiology of schizophrenia^27^ have informed the development of biomarkers. Neurochemical imaging with positron emission tomography (PET) consistently shows an association between elevated dopamine synthesis capacity in the striatum, preferentially in the associative portion,^52^ and symptom severity of psychosis.^53^ Also, similar studies relate the clinical efficacy of antipsychotic drugs to the postsynaptic dopamine 2/3 receptor modulation within a critical window.^54^ Functional MRI (fMRI) data suggest aberrant striatal functional connectivity in relation to symptom severity and normalization of such baseline aberrant functional connectivity with antipsychotic treatment response.^55^, ^56^ Similarly, studies of fMRI signal in the striatum in response to reward anticipation also suggest such a pattern of blunted response at baseline that is corrected along with treatment response.^57^, ^58^ These advances in understanding the pathophysiology of the illness have supported the development of prognostic neuroimaging biomarkers. For instance, Sarpal and colleagues developed an fMRI measure of functional connectivity, known as the Striatal Connectivity Index (SCI), as a prognostic biomarker of treatment response.^55^ In a cohort of 41 individuals with first episode schizophrenia receiving 12 weeks of antipsychotic medication, lower SCI values prior to treatment onset, reflecting more aberrant striatal resting state functional connectivity, were associated with treatment response, predicting it with ~80% sensitivity and specificity, which was replicated in an independent cohort. Further validity of the SCI has been provided by its application to study the effects of cannabis use on treatment response in schizophrenia.^59^ Most recently, the development of prognostic biomarkers for treatment response in the context of psychotic relapse has shown promise.^60^ The ultimate goal of this research is developing biomarkers of relapse risk that facilitate decision‐making about long‐term treatment, for example to inform whether antipsychotics should be maintained long‐term after remission for a given individual.^61^

## FROM ASSUMING THAT A PRESCRIPTION RESULTS IN THE DRUG REACHING THE BRAIN, TO ADDRESSING THE CHALLENGES OF CONTINUOUS TREATMENT ADHERENCE

It has been recognized for a long time that taking medications as prescribed is a major challenge in all areas of medicine.^62^ Medication use behavior that deviates from the prescriber's recommendations is observed in as many as 50% of medication users at any given time. Of all medication‐related hospital admissions in the United States, 33%–69% are due to poor medication adherence, with a resultant cost of approximately $100 billion a year.^63^, ^64^ The factors influencing medication nonadherence are complex and include insight, lifestyle, psychological well‐being, health literacy, support systems, and side effects of medications.^65^ In schizophrenia, issues like lack of insight, insufficient social support, cognitive symptoms, or side effects of medication play a negative role in the ability to be adherent with antipsychotic maintenance over the long term. This is worrisome, since nonadherence with antipsychotic medication is the strongest risk factor of relapse in schizophrenia.^21^

Depending on how it is measured, nonadherence is present in between 10% and 60% of individuals receiving pharmacological treatment for schizophrenia at any given time.^66^ Circumstances that increase the risk for nonadherence in schizophrenia are being at an earlier phase of treatment,^67^, ^68^ comorbid substance use,^69^ social exclusion (i.e., homelessness), and high side effect burden.^66^ The assessment of nonadherence with oral medications is indeed a challenge, with no method that is clearly reliable and scalable to routine practice. For instance, antipsychotic plasma levels are probably the most reliable method to provide at least cross‐sectional data on adherence, but measurement of these is not yet routine in most healthcare systems. Alternatively, pharmacy records or self‐reports are easier to obtain in clinical practice but are less reliable. A study of clinician adherence assessment for patients who were brought to an emergency room because of acute psychosis revealed that when checked against plasma levels, the clinician's routine assessment of adherence was not much better than chance. In this sample, only one‐third of individuals had therapeutic concentrations of antipsychotic drugs in plasma, and only four in 10 who were nonadherent were correctly identified by clinicians.^70^ These data highlight how even when identifying treatment adherence status is most critical, such as in an emergency caused by acute psychosis, clinician assessment is very unreliable, very often by overestimating treatment adherence.

How can this important challenge in the treatment of schizophrenia be addressed? We have learnt important lessons. One is that we should normalize nonadherence rather than stigmatizing it. As evident also from other areas of medicine, taking oral medications on a regular basis is challenging for many reasons. It is much more productive to try to understand these reasons rather than label this as “bad behavior.” Very likely, a frank conversation may highlight important issues that should be discussed and addressed in the context of recovery‐oriented care. Second, these conversations can only occur if the prescriber asks explicitly about them. Unfortunately, asking about adherence is less frequent than it should be, probably because many clinicians often overestimate adherence rates.^66^, ^71^ Third and most importantly, evidence‐based interventions should be used to mitigate nonadherence. Although several options, including a medication event monitoring system (MEMS), tablets that can register when they are ingested,^72^ or digital applications that can document when a pill is ingested,^73^ the most reliable and scalable option to address nonadherence is use of an LAI antipsychotic. In our opinion, there are few reasons not to use an LAI formulation whenever an antipsychotic is necessary over the mid‐ to long‐term. There are robust data indicating that LAIs are superior to their oral counterparts in preventing hospitalization. For instance, within‐individual participant analyses of treatment utilization and hospitalization for psychosis in a Swedish national cohort concluded that, for a given individual, treatment periods on LAIs may be about 30% more effective than treatment periods on their oral counterparts in delaying hospitalization.^74^ This finding has been consistently replicated in mirror‐image study and epidemiological sample meta‐analyses. Data from randomized clinical trials (RCTs) are less consistent, but also supportive.^75^ It is very likely that inconsistency in RCTs is related to individuals agreeing to participate in clinical trials being less at risk of treatment nonadherence than those who are represented in epidemiological datasets.^76^ In addition, the clinical trial itself changes the ecology of care and might promote better adherence in the control condition than usual care. In addition to the data on preventing relapse and hospitalization, there are data suggesting that LAI treatment episodes, compared to treatment episodes of oral antipsychotics, take longer to be discontinued.^77^ This benefit may be particularly important for individuals in the earlier phases of treatment, who are much more likely to interrupt treatment and experience a subsequent relapse.^67^, ^68^ Another advantage of LAIs is related to mortality. Given the much shorter life expectancy for people with schizophrenia,^32^ and the known overall association between greater use of antipsychotic medication and longer life expectancy in schizophrenia, it is hypothesized, with some data supporting,^33^ that by guaranteeing continuous drug delivery and being used for longer periods of time compared to their oral counterparts, the use of LAIs is associated with longer life expectancy than the use of oral antipsychotics. Finally, another potential advantage of LAIs is that by guaranteeing drug delivery, they may limit the impact of the risk factors of relapse that are mediated through nonadherence. For instance, co‐occurring substance use and treatment nonadherence are both risk factors of relapse in schizophrenia,^21^ and it is deemed that about one‐third of the variance of co‐occurring substance use in relapse is mediated through interrupting antipsychotic treatment rather than through the direct effects of drugs of abuse in the brain.^69^ It is possible that by assuring continuous treatment delivery, LAIs mitigate some of the risk of relapse associated with co‐occurring substance use. Regarding potential disadvantages of LAIs compared to oral antipsychotics, a meta‐analysis did not find many meaningful differences in terms of side effects between LAIs and oral antipsychotics,^78^ and recent data on neuroleptic malignant syndrome, probably one of the most serious side effects of antipsychotic treatment, showed longer time to recovery but no differences in morbimortality for individuals who were on LAIs compared to those who were on oral drugs.^79^, ^80^

These data suggest that LAIs should probably be used from the early phase of treatment since this is one of the periods with greater risk of treatment nonadherence,^67^, ^68^ and because preventing relapses early in the course of illness may mitigate their impact on social and occupational functioning in these young individuals. Traditionally LAIs have been used after several relapses, when they may have less of a long‐term impact.^81^ However, there is growing recognition of the importance of making LAIs the preferred formulation for the treatment of schizophrenia in earlier phase treatment, before several relapses have occurred. Recently, the PRELAPSE trial demonstrated that LAIs are well accepted in early phases psychosis individuals, with about 90% of those for whom the LAI formulation was offered taking at least one injection.^71^ The main intervention of this cluster‐randomized trial was training clinicians on how to talk to patients about LAIs in the experimental clinics as compared to those in the clinics delivering usual care. Emphasis was made in focusing on the potential benefits of LAIs rather than the method of delivery (i.e., injection). In addition, the immediate advantages in the life of the user were emphasized (i.e., more convenient than taking pills every day, no daily reminder of the illness, no confrontation with caregivers or prescriber about whether treatment is being used properly, and so forth). Furthermore, misconceptions about LAIs (i.e., the drug is controlling me rather than treatment allows me to control my symptoms, injections are very painful, I will not be able to stop) were addressed. Addressing these crucial elements to make a high‐quality presentation of LAIs in fact translates into better clinical outcomes. In the PRELAPSE study, individuals in the early phase of schizophrenia receiving treatment in clinics who were trained on these issues were significantly less likely to be hospitalized over follow‐up than those who received treatment under usual care conditions.^82^

Unfortunately, despite the advantages of LAIs guaranteeing treatment delivery in the relapse‐prevention phase of illness, some individuals will relapse despite ongoing treatment. This is known as breakthrough on antipsychotic maintenance medication (BAMM).^83^ This phenomenon has been challenging to study, given the confounder of nonadherence in relapse in schizophrenia. As discussed above, nonadherence is the strongest predictor of relapse in schizophrenia.^21^ It is very common, with as many as half of the individuals at any given time not taking medicine as prescribed.^66^ It has been difficult to determine the actual contribution of nonadherence to relapse since there are no scalable means to reliably quantify it. In these circumstances, the use of LAI antipsychotics is indeed convenient, since actual drug exposure can be easily and reliably quantified at the time of relapse with the record of injection administration, thus removing the confounder of nonadherence. Using this approach, data have suggested that relapse despite ongoing antipsychotic treatment is relatively common. In an individual participant data meta‐analysis of time to relapse among individuals randomized to an LAI in relapse‐prevention clinical trials, there were almost 25 relapse events per 100 participant years of LAI continuous treatment.^84^ In this study, presence of tardive dyskinesia was the strongest predictor of relapse despite ongoing treatment, pointing out a possibly shared mechanisms between the pathophysiology of tardive dyskinesia and relapse in psychosis. BAMM was also studied in a national Scandinavian cohort of individuals with schizophrenia treated with LAIs over up to 20 years, with about one in three of those treated with an LAI experiencing relapse at some point in their lives despite ongoing treatment.^85^

## FROM LACK OF GUIDANCE FOR MAINTENANCE TREATMENT, TO EVIDENCE‐BASED RECOMMENDATIONS

Once there has been success in treating acute psychotic symptoms with antipsychotic drugs, the next important question is, what dose should be used and for how long? Most of those treating or living with schizophrenia, as well as families have asked this question at some point. There has been an accumulation of data over the last several decades that facilitate answering this important question.

Two separate meta‐analyses have addressed the question of dosing of antipsychotic maintenance medication for relapse prevention, with similar conclusions. One of them compared relapse rates between individuals randomized to standard or low (i.e., <50% of standard) dose, across 24 trials and 3282 individuals. Compared with the standard dose, a low dose increased the risk of relapse by 44% and the risk of all‐cause discontinuation by 12%.^86^ Similarly, a frequentist dose–response meta‐analysis of 72 dose arms, corresponding to 4776 individuals, of randomized controlled trials of antipsychotics for relapse preventions in schizophrenia found hyperbolic efficacy‐related dose–response curves, meaning that the risk of relapse decreased rapidly with increasing dose of antipsychotics during maintenance, up to an equivalent of 5 mg of risperidone, above which there was no increased benefit in relapse prevention but increasing rates of side effects. Interestingly, the analysis of studies who recruited individuals in remission of psychotic symptoms, showed the curve bent earlier, suggesting that equivalents of 2.5 mg of risperidone may be the optimal balance between efficacy and potential side effects among individuals who sustain full remission (generally in the studies selected mild or fewer symptoms for longer than 1 year).^87^ Thus, it can be concluded from this body of research that unless there has been sustained remission of symptoms, it is generally recommended to continue with the same dose of antipsychotics that was used for symptom stabilization.

Regarding how long maintenance treatment should be continued, the randomized controlled data for up to 3 years after acute psychosis shows clear superiority of antipsychotic maintenance compared to treatment discontinuation in preventing relapse and treatment disengagement.^88^ It has been observed that the effect size for the advantages of antipsychotic continuation decrease with longer studies.^88^ Also, some uncontrolled and/or unrandomized data have shown better recovery outcomes in individuals who discontinue antipsychotics over the long term compared to those who continue treatment.^89^, ^90^ Altogether, these data questioned whether the benefit of maintenance treatment would dissipate over time or, as has been proposed by some, whether it could have long‐term detrimental effects.^91^ These theories have been hypothesized because of antipsychotic withdrawal, which would result in rebound psychosis,^92^ but this theory has not been validated by either clinical research of abrupt versus progressive treatment discontinuation,^88^ nor neurobiological research on dopamine receptor occupancy in response to antipsychotic discontinuation and risk of relapse.^93^ In our opinion,^94^ in agreement with other experts,^95^ there is insufficient data to suggest that the benefits of antipsychotic drugs would disappear over time or that they would indeed become harmful. For instance, it is very likely that the decreasing effect size of antipsychotics in maintenance treatment over time in randomized controlled studies is related to accumulating nonadherence in the treatment continuation arms, which would result in less difference between antipsychotic and placebo. Also, the long‐term cohort studies that have been most often used to argue for long‐term detrimental effects of antipsychotics are highly exposed to confounder by indication, meaning that those with better prognosis would self‐select to the treatment discontinuation group, and those with worse prognosis would be more likely to be in the antipsychotic maintenance group (i.e., reverse causation). Similarly, data from a highly cited clinical trial of dose reduction/treatment discontinuation versus maintenance treatment for up to 7 years, in which recovery outcomes were better for the dose reduction/treatment discontinuation group,^90^ is inconclusive given the low representativeness of the baseline cohort of individuals with schizophrenia (as opposed to psychosis not otherwise specified or schizophreniform disorder), the exposure to confounder by indication since individuals and prescribers could decide whether to stay on dose maintenance or treatment discontinuation, and by the fact that there was not an effective difference in treatment exposure between the two groups, both of which differed only in 1 mg equivalent of haloperidol. In fact, the analysis of this dataset for the initial 2 years, while individuals could not self‐select to either group depending on their illness severity, shows significantly better outcomes for those who remained on treatment than for those who discontinued.^96^ In our interpretation, given the serious validity concerns about the data supporting long‐term discontinuation of antipsychotic maintenance treatment after the initial 2–3 years, and the robustness of the data supporting antipsychotic maintenance prior to that time point, we would argue against an indiscriminate discontinuation of long‐term antipsychotic medication.

Nevertheless, a small minority of individuals can recover without antipsychotic maintenance treatment.^97^ The question is whether the risk of relapse following antipsychotic discontinuation can be determined at the level of the individual, so that informed decisions can be made about for whom it would be recommendable to discontinue treatment. Unfortunately, most clinical predictors of successful treatment discontinuation have not been replicated, except for history of relapse following previous treatment discontinuation,^97^ which leaves trial and error as the only method to determine for whom antipsychotic discontinuation may be suitable. Given the short‐ and long‐term impacts of relapse in schizophrenia, ranging from potential risk to self or others to lower chance of treatment responsiveness on relapse, we thus recommend caution with regard to when and for whom to discontinue antipsychotic maintenance treatment.

## FROM DOPAMINE TYPE 2 RECEPTOR BLOCKADE HEGEMONY TO NOVEL MECHANISMS OF ACTION

One of the main criticisms of the progress in the psychopharmacology of schizophrenia has been the lack of novel mechanisms of action for antipsychotic drugs since the first antipsychotics were synthesized in the 1950s, and a surge of “me too” new drugs without clear advantages over already approved agents.^98^ Until recently, the most relevant progress was the development of second‐generation antipsychotic drugs in the 1980s, which in addition to having dopaminergic modulation also had serotoninergic effects, which are not considered as mechanistic towards antipsychotic efficacy but may result in a more favorable tolerability profile,^99^ as well as the so‐called “third generation,” such as aripiprazole, brexpiprazole or cariprazine, which are partial agonists, rather than blockers of the dopamine 2/3 receptor.^100^ At the same time, we are still uncertain as to the mechanistic reasons for clozapine's established superiority in treatment‐resistant schizophrenia (TRS) patients, despite many years of availability. We seem to be now living in a pivotal moment in this regard, with promising results in early phase studies of drugs that do not rely on direct dopaminergic receptor modulation. In particular, xanomeline,^101^ a muscarinic agonist, and SEP‐363856,^102^ a trace amine‐associated receptor 1 (TAAR 1) agonist, have gone through phase 2 studies with promising results.

Drugs with muscarinic effects have been targeted as potential antipsychotic agents, with experimentation that dates back to the 1950s with anti‐ and pro‐muscarinic drugs,^103^ although given the problems with tolerability, particularly peripheral effects, this line of research was abandoned for decades. Most recently, interest in the contribution of neural circuits modulated by muscarinic receptors to the pathophysiology of schizophrenia has surged,^104^, ^105^ and with it the experimentation of muscarinic agonists as antipsychotic drugs. The agent that so far has made the most progress is xanomeline, which has been tested in combination with trospium, a peripheral anticholinergic agent, to mitigate the peripheral pro‐cholinergic effects that would limit acceptability. In a phase 2 5‐week, double‐blind placebo‐controlled randomized trial, xanomeline‐trospium had a statistically significant difference with placebo of −5.9 points in total psychopathology measured with the positive and negative syndrome scale (PANSS), with secondary endpoints aligning with the main outcome.^101^ Of note, the most relevant side effects were peripheral anticholinergic, but they were modest and other adverse events were minimal. These promising results have substantiated further research, with four phase 3 clinical trials underway.^106^

The other promising compound with a novel mechanism of action is SEP‐363856, a TAAR‐1 agonist. TAAR‐1 are intracellular receptors expressed widely in peripheral organs, which in the central nervous system are particularly expressed in monoaminergic neurons. One of the effects of this intracellular receptor is to regulate presynaptic dopamine release capacity. In particular, TAAR‐1 agonists effectively decrease the firing rate of mesolimbic dopaminergic neurons, which have been involved in the pathophysiology of schizophrenia. In a phase 2 placebo‐controlled randomized clinical trial, SEP‐363856, a TAAR‐1 agonist, demonstrated superiority compared to placebo, with a difference with placebo of −7.9 points in total psychopathology measured with the PANSS. Interestingly, there was a statistical trend (which did not survive significance correction for multiple comparisons) that favored this compound in global assessment of functioning, as well as in negative symptoms. Another important consideration is that this compound did not seem to have greater effects on extrapyramidal symptoms and changes in lipid profile, glycated hemoglobin, and prolactin than placebo.^102^ Thus, this compound has shown promise related to a novel mechanism of action and a tolerability profile that would provide added value to the antipsychotics currently available in the market. Currently, there are five phase 3 clinical trials for SEP‐363856 which are expected to provide results by the end of 2022, beginning of 2023.^107^

## THE ROAD AHEAD

The progress in the treatment of schizophrenia described here has been an iterative process of identifying areas of concern, developing interventions that showed promise under optimal circumstances, and finally rolling them out to real‐world settings. Some of the progress described here has been the result of fulfilling this entire cycle, whereas in other areas more work may be necessary in order to translate the identification of areas of concern into better outcomes for those living with schizophrenia in the community. In fact, there are some areas for which we still struggle to generate interventions that would show efficacy under optimal testing circumstances, specifically negative or cognitive symptoms. A broad range of pharmacological interventions, including antidepressant,^108^ stimulant^109^ drugs in augmentation of antipsychotics, have been tested with limited success. Another area of concern are residual symptoms despite clozapine treatment. To date we do not have effective interventions for clozapine treatment failures besides adjunctive ECT and possibly aripiprazole.^110^, ^111^ These are examples of critical barriers in developing therapeutics that hopefully will be better addressed along with discoveries on their pathophysiological underpinnings. However, we believe that for many of the areas of need, there are interventions that have already shown promise under optimal testing circumstances, but that have not yet been rolled out to the real world. We would argue that the most relevant examples are clozapine and long‐acting injectables. The data supporting the effectiveness of these drugs is as robust as it gets in psychiatry research, nonetheless they are widely underutilized in the field. For instance, despite the prevalence of treatment‐resistant schizophrenia being one in three, in the United States the utilization rate of clozapine (the only drug with regulatory approval for this indication) is between 1% and 11% of those receiving antipsychotic treatment for schizophrenia.^49^ Similarly, LAIs have been consistently associated with longer time to treatment discontinuation,^68^ lower risk of rehospitalization,^75^ and even lower mortality rates,^33^ with no disadvantage compared to their oral counterparts,^78^ yet in the United States their utilization rate is between 4% and 22%.^49^ Thus, the road ahead should involve innovation in drug development to target the pathophysiology of the illness more directly in ways previously unaddressed, but very importantly to also develop strategies to overcome the barriers to the utilization of interventions that have already demonstrated superior effectiveness.

## AUTHOR CONTRIBUTIONS

John M. Kane and Jose M. Rubio have contributed equally to the manuscript.

## CONFLICTS OF INTEREST

J.R. has received honoraria from TEVA pharmaceuticals and Janssen, royalties from UpToDate, and grant support from Alkermes. J.K. has received honoraria from Alkermes, Allergan, Dainippon Sumitomo, H. Lundbeck, Indivior, Intracellular Therapies, Janssen Pharmaceutical, Johnson & Johnson, LB Pharmaceuticals, Merck, Minerva, Neurocrine, Novartis Pharmaceuticals, Otsuka, Reviva, Roche, Saladex, Sunovion, Takeda, Teva; grant support from Otsuka, Lundbeck, Sunovion, and Janssen; is a shareholder of the Vanguard Research Group, LB Pharmaceuticals Inc., and North Shore Therapeutics; and receives royalties from UptoDate.

## ETHICS APPROVAL STATEMENT

Ethics approval was not warranted since no new data was collected for this manuscript.

## Data Availability

No new data was presented in this manuscript.

## References

[1] McCutcheon RA , Reis Marques T , Howes OD . Schizophrenia—an overview. JAMA Psychiatry. 2019; 77(2):201–10. 10.1001/jamapsychiatry.2019.3360 31664453

[2] GBD Project Collaborators . Global, regional, and national incidence, prevalence, and years lived with disability for 328 diseases and injuries for 195 countries, 1990–2016: a systematic analysis for the Global Burden of Disease Study 2016. Lancet. 2017;390(10100):1211–59. 10.1016/S0140-6736(17)32154-2 28919117 PMC5605509

[3] Crump C , Winkleby MA , Sundquist K , Sundquist J . Comorbidities and mortality in persons with schizophrenia: a Swedish national cohort study. Am J Psychiatry. 2013;170(3):324–33. 10.1176/appi.ajp.2012.12050599 23318474

[4] Leucht S , Leucht C , Huhn M , Chaimani A , Mavridis D , Helfer B , et al. Sixty years of placebo‐controlled antipsychotic drug trials in acute schizophrenia: systematic review, bayesian meta‐analysis, and meta‐regression of efficacy predictors. Am J Psychiatry. Published online May 25 2017; 174(10):927–42. 10.1176/appi.ajp.2017.16121358 28541090

[5] Anthony WA . A recovery‐oriented service system: setting some system level standards. Psychiatr Rehabil J. 2000;24(2):159–68. 10.1037/h0095104

[6] Jääskeläinen E , Juola P , Hirvonen N , McGrath JJ , Saha S , Isohanni M , et al. A systematic review and meta‐analysis of recovery in schizophrenia. Schizophr Bull. 2013;39(6):1296–306. 10.1093/schbul/sbs130 23172003 PMC3796077

[7] McGorry PD , Yung AR . Early intervention in psychosis: an overdue reform. Aust N Z J Psychiatry. 2003;37(4):393–8. 10.1046/j.1440-1614.2003.01192.x 12873322

[8] Birchwood M , Todd P , Jackson C . Early intervention in psychosis: the critical period hypothesis. Br J Psychiatry. 1998;172(S33):53–9. 10.1192/S0007125000297663 9764127

[9] Dixon L . What it will take to make coordinated specialty care available to anyone experiencing early schizophrenia: getting over the hump. JAMA Psychiatry. 2017;74(1):7–8. 10.1001/jamapsychiatry.2016.2665 27851843

[10] Kane JM , Schooler NR , Marcy P , Correll CU , Brunette MF , Mueser KT , et al. The RAISE early treatment program for first‐episode psychosis: background, rationale, and study design. J Clin Psychiatry. 2015;76(3):240–6. 10.4088/JCP.14m09289 25830446 PMC7477907

[11] Kane JM , Robinson DG , Schooler NR , Mueser KT , Penn DL , Rosenheck RA , et al. Comprehensive versus usual community care for first‐episode psychosis: 2‐year outcomes from the NIMH RAISE Early Treatment Program. Am J Psychiatry. 2016;173(4):362–72. 10.1176/appi.ajp.2015.15050632 26481174 PMC4981493

[12] Correll CU , Galling B , Pawar A , Krivko A , Bonetto C , Ruggeri M , et al. Comparison of early intervention services vs treatment as usual for early‐phase psychosis: a systematic review, meta‐analysis, and meta‐regression. JAMA Psychiatry. 2018;75(6):555–65. 10.1001/jamapsychiatry.2018.0623 29800949 PMC6137532

[13] Robinson DG , Schooler NR , Correll CU , John M , JKurien BT , Marcy P , et al. Psychopharmacological treatment in the RAISE‐ETP study: outcomes of a manual and computer decision support system based intervention. Am J Psychiatry. 2017; 175(2):169–79. appiajp201716080919 10.1176/appi.ajp.2017.16080919 28945118 PMC5794655

[14] Robinson DG , Schooler NR , John M , Correll CU , Marcy P , Addington J , et al. Prescription practices in the treatment of first‐episode schizophrenia spectrum disorders: data from the national RAISE‐ETP study. AJP. 2015;172(3):237–48. 10.1176/appi.ajp.2014.13101355 PMC4358323 25727536

[15] Reist C , Valdes E , Ren Y , Wright A , Rubio JM . Using claims data to assess treatment quality of first‐episode psychosis. PS (Wash DC). 2021;72(3):247–53. 10.1176/appi.ps.201900595 33167819

[16] Westfall JM , Mold J , Fagnan L . Practice‐based research—“Blue Highways” on the NIH roadmap. JAMA. 2007;297(4):403–6. 10.1001/jama.297.4.403 17244837

[17] Hunt GE , Large MM , Cleary M , Lai HMX , Saunders JB . Prevalence of comorbid substance use in schizophrenia spectrum disorders in community and clinical settings, 1990–2017: systematic review and meta‐analysis. Drug Alcohol Depend. 2018;191:234–58. 10.1016/j.drugalcdep.2018.07.011 30153606

[18] Swofford CD , Kasckow JW , Scheller‐Gilkey G , Inderbitzin LB . Substance use: a powerful predictor of relapse in schizophrenia. Schizophr Res. 1996;20(1–2):145–51. 10.1016/0920-9964(95)00068-2 8794502

[19] Dixon L , Postrado L , Delahanty J , Fischer PJ , Lehman A . The association of medical comorbidity in schizophrenia with poor physical and mental health. J Nerv Ment Dis. 1999;187(8):496–502. 10463067 10.1097/00005053-199908000-00006

[20] Rosen CS , Kuhn E , Greenbaum MA , Drescher KD . Substance abuse‐related mortality among middle‐aged male VA psychiatric patients. PS (Wash DC). 2008;59(3):290–6. 10.1176/ps.2008.59.3.290 18308910

[21] Alvarez‐Jimenez M , Priede A , Hetrick SE , Bendall S , Killackey E , Parker AG , et al. Risk factors for relapse following treatment for first episode psychosis: a systematic review and meta‐analysis of longitudinal studies. Schizophr Res. 2012;139(1–3):116–28. 10.1016/j.schres.2012.05.007 22658527

[22] Kerfoot KE , Rosenheck RA , Petrakis IL , Swartz MS , Keefe RS , McEvoy JP , et al. Substance use and schizophrenia: adverse correlates in the CATIE study sample. Schizophr Res. 2011;132(2–3):177–82. 10.1016/j.schres.2011.07.032 21872443

[23] Dixon L . Dual diagnosis of substance abuse in schizophrenia: prevalence and impact on outcomes. Schizophr Res. 1999;35(Suppl):S93–100. 10.1016/S0920-9964(98)00161-3 10190230

[24] Dubol M , Trichard C , Leroy C , Sandu AL , Rahim M , Granger B , et al. Dopamine transporter and reward anticipation in a dimensional perspective: a multimodal brain imaging study. Neuropsychopharmacology. 2018;43(4):820–7. 10.1038/npp.2017.183 28829051 PMC5809789

[25] Kapur S . Psychosis as a state of aberrant salience: a framework linking biology, phenomenology, and pharmacology in schizophrenia. Am J Psychiatry. 2003;160(1):13–23. 10.1176/appi.ajp.160.1.13 12505794

[26] Volkow ND . Substance use disorders in schizophrenia – clinical implications of comorbidity. Schizophr Bull. 2009;35(3):469–72. 10.1093/schbul/sbp016 19325163 PMC2669586

[27] Maia TV , Frank MJ . An integrative perspective on the role of dopamine in schizophrenia. Biol Psychiatry. 2017;81(1):52–66. 10.1016/j.biopsych.2016.05.021 27452791 PMC5486232

[28] World Health Organization . Statistics on tobacco use. Available from: https://www.who.int/news-room/fact-sheets/detail/tobacco. Accessed 1 April 2022.

[29] Oluwoye O , Monroe‐DeVita M , Burduli E , Chwastiak L , McPherson S , McClellan JM , et al. Impact of tobacco, alcohol and cannabis use on treatment outcomes among patients experiencing first episode psychosis: data from the national RAISE‐ETP study. Early Interv Psychiatry. 2019;13(1):142–6. 10.1111/eip.12542 29356438 PMC6684200

[30] Centers for Disease Control . CDC report on tobacco use in the United States. Available from: https://www.cdc.gov/tobacco/campaign/tips/resources/data/cigarette-smoking-in-united-states.html?s_cid=OSH_tips_GL0005&utm_source=google&utm_medium=cpc&utm_campaign=TipsRegular+2021%3BS%3BWL%3BBR%3BIMM%3BDTC%3BCO&utm_content=Smoking+-+Facts_P&utm_term=statistics+about+smoking&gclid=CjwKCAiAvriMBhAuEiwA8Cs5lZ4-FnBfDNsdegdnN04sBxlsU5w0maLDe4LlGy8iKoe2z0G5m5lXMhoChsYQAvD_BwE&gclsrc=aw.ds. Accessed 1 April 2022.

[31] Olfson M , Gerhard T , Huang C , Crystal S , Stroup TS . Premature mortality among adults with schizophrenia in the United States. JAMA Psychiatry. 2015;72(12):1172–81. 10.1001/jamapsychiatry.2015.1737 26509694

[32] Saha S , Chant D , McGrath J . A systematic review of mortality in schizophrenia: is the differential mortality gap worsening over time? Arch Gen Psychiatry. 2007;64(10):1123–31. 10.1001/archpsyc.64.10.1123 17909124

[33] Taipale H , Mittendorfer‐Rutz E , Alexanderson K , Majak M , Mehtälä J , Hoti F , et al. Antipsychotics and mortality in a nationwide cohort of 29,823 patients with schizophrenia. Schizophr Res. 2018;197:274–80. 10.1016/j.schres.2017.12.010 29274734

[34] Vancampfort D , Stubbs B , Mitchell AJ , De Hert M , Wampers M , Ward PB , et al. Risk of metabolic syndrome and its components in people with schizophrenia and related psychotic disorders, bipolar disorder and major depressive disorder: a systematic review and meta‐analysis. World Psychiatry. 2015;14(3):339–47. 10.1002/wps.20252 26407790 PMC4592657

[35] Chang SC , Goh KK , Lu ML . Metabolic disturbances associated with antipsychotic drug treatment in patients with schizophrenia: state‐of‐the‐art and future perspectives. World J Psychiatry. 2021;11(10):696–710. 10.5498/wjp.v11.i10.696 34733637 PMC8546772

[36] Cohn TA , Sernyak MJ . Metabolic monitoring for patients treated with antipsychotic medications. Can J Psychiatry. 2006;51(8):492–501. 10.1177/070674370605100804 16933586

[37] Fiorillo A , Barlati S , Bellomo A , Corrivetti G , Nicolò G , Sampogna G , et al. The role of shared decision‐making in improving adherence to pharmacological treatments in patients with schizophrenia: a clinical review. Ann Gen Psychiatry. 2020;19(1):43. 10.1186/s12991-020-00293-4 32774442 PMC7409631

[38] Chien WT , Mui JH , Cheung EF , Gray R . Effects of motivational interviewing‐based adherence therapy for schizophrenia spectrum disorders: a randomized controlled trial. Trials. 2015;16(1):270. 10.1186/s13063-015-0785-z 26072311 PMC4469254

[39] Collins FS , Varmus H . A new initiative on precision medicine. N Engl J Med. 2015;372(9):793–5. 10.1056/NEJMp1500523 25635347 PMC5101938

[40] Carbon M , Hsieh CH , Kane JM , Correll CU . Tardive dyskinesia prevalence in the period of second‐generation antipsychotic use: a meta‐analysis. J Clin Psychiatry. 2017;78(3):e264–78. 10.4088/JCP.16r10832 28146614

[41] Kalia M . Biomarkers for personalized oncology: recent advances and future challenges. Metabolism. 2015;64(3 Suppl 1):S16–21. 10.1016/j.metabol.2014.10.027 25468140

[42] Kane J , Honigfeld G , Singer J , Meltzer H . Clozapine for the treatment‐resistant schizophrenic. A double‐blind comparison with chlorpromazine. Arch Gen Psychiatry. 1988;45(9):789–96. 3046553 10.1001/archpsyc.1988.01800330013001

[43] Kane JM , Correll CU . The role of clozapine in treatment‐resistant schizophrenia. JAMA Psychiatry. 2016;73(3):187–8. 10.1001/jamapsychiatry.2015.2966 26841681

[44] Howes OD , Kapur S . A neurobiological hypothesis for the classification of schizophrenia: type A (hyperdopaminergic) and type B (normodopaminergic). Br J Psychiatry. 2014;205(1):1–3. 10.1192/bjp.bp.113.138578 24986384

[45] Howes OD , McCutcheon R , Agid O , de Bartolomeis A , van Beveren NJ , Birnbaum ML , et al. Treatment‐resistant schizophrenia: Treatment Response and Resistance in Psychosis (TRRIP) working group consensus guidelines on diagnosis and terminology. Am J Psychiatry. 2017;174(3):216–29. 10.1176/appi.ajp.2016.16050503 27919182 PMC6231547

[46] Kahn RS , Winter van Rossum I , Leucht S , McGuire P , Lewis SW , Leboyer M , et al. Amisulpride and olanzapine followed by open‐label treatment with clozapine in first‐episode schizophrenia and schizophreniform disorder (OPTiMiSE): a three‐phase switching study. Lancet Psychiatry. 2018;5(10):797–807. 10.1016/S2215-0366(18)30252-9 30115598

[47] Homan P , Kane JM . Clozapine as an early‐stage treatment. Acta Psychiatr Scand. 2018;138(4):279–80. 10.1111/acps.12965 30240545

[48] Bachmann CJ , Aagaard L , Bernardo M , Brandt L , Cartabia M , Clavenna A , et al. International trends in clozapine use: a study in 17 countries. Acta Psychiatr Scand. 2017;136(1):37–51. 10.1111/acps.12742 28502099

[49] Bareis N , Olfson M , Wall M , Stroup TS . Variation in psychotropic medication prescription for adults with schizophrenia in the United States. Psychiatr Serv. 2021;73(5):492–500. 10.1176/appi.ps.202000932 34587788 PMC8964836

[50] Samara MT , Leucht C , Leeflang MM , Anghelescu IG , Chung YC , Crespo‐Facorro B , et al. Early improvement as a predictor of later response to antipsychotics in schizophrenia: a diagnostic test review. Am J Psychiatry. 2015;172(7):617–29. 10.1176/appi.ajp.2015.14101329 26046338

[51] Remington G , Addington D , Honer W , Ismail Z , Raedler T , Teehan M . Guidelines for the pharmacotherapy of schizophrenia in adults. Can J Psychiatry. 2017;62(9):604–16. 10.1177/0706743717720448 28703015 PMC5593252

[52] McCutcheon R , Beck K , Jauhar S , Howes OD . Defining the locus of dopaminergic dysfunction in schizophrenia: a meta‐analysis and test of the mesolimbic hypothesis. Schizophr Bull. 2018;44(6):1301–11. 10.1093/schbul/sbx180 29301039 PMC5933516

[53] Howes OD , Kambeitz J , Kim E , Stahl D , Slifstein M , Abi‐Dargham A , et al. The nature of dopamine dysfunction in schizophrenia and what this means for treatment. Arch Gen Psychiatry. 2012;69(8):776–86. 10.1001/archgenpsychiatry.2012.169 22474070 PMC3730746

[54] Uchida H , Takeuchi H , Graff‐Guerrero A , Suzuki T , Watanabe K , Mamo DC . Dopamine D2 receptor occupancy and clinical effects: a systematic review and pooled analysis. J Clin Psychopharmacol. 2011;31(4):497–502. 10.1097/JCP.0b013e3182214aad 21694629

[55] Sarpal DK , Argyelan M , Robinson DG , Szeszko PR , Karlsgodt KH , John M , et al. Baseline striatal functional connectivity as a predictor of response to antipsychotic drug treatment. Am J Psychiatry. 2016;173(1):69–77. 10.1176/appi.ajp.2015.14121571 26315980 PMC4845897

[56] Li A , Zalesky A , Yue W , Howes O , Yan H , Liu Y , et al. A neuroimaging biomarker for striatal dysfunction in schizophrenia. Nat Med. 2020;26(4):558–65. 10.1038/s41591-020-0793-8 32251404

[57] Nielsen MØ , Rostrup E , Wulff S , Bak N , Lublin H , Kapur S , et al. Alterations of the brain reward system in antipsychotic naïve schizophrenia patients. Biol Psychiatry. 2012;71(10):898–905. 10.1016/j.biopsych.2012.02.007 22418013

[58] Nielsen MO , Rostrup E , Wulff S , Bak N , Broberg BV , Lublin H , et al. Improvement of brain reward abnormalities by antipsychotic monotherapy in schizophrenia. Arch Gen Psychiatry. 2012;69(12):1195–204. 10.1001/archgenpsychiatry.2012.847 22868877

[59] Blair Thies M , DeRosse P , Sarpal DK , et al. Interaction of cannabis use disorder and striatal connectivity in antipsychotic treatment response. Schizophrenia Bulletin Open. 2020;1(1):sgaa014. 10.1093/schizbullopen/sgaa014 32803161 PMC7418867

[60] Rubio JM , Lencz T , Barber A , Moyett A , Ali S , Bassaw F , et al. Striatal functional connectivity in psychosis relapse: a hypothesis generating study. Schizophr Res. 2021;S0920‐9964(21):00222. 10.1016/j.schres.2021.06.010 34183210

[61] Rubio JM , Malhotra AK , Kane JM . Towards a framework to develop neuroimaging biomarkers of relapse in schizophrenia. Behav Brain Res. 2021;402:113099. 10.1016/j.bbr.2020.113099 33417996

[62] Osterberg L , Blaschke T . Adherence to medication. N Engl J Med. 2005;353(5):487–97. 10.1056/NEJMra050100 16079372

[63] Senst BL , Achusim LE , Genest RP , Cosentino LA , Ford CC , Little JA , et al. Practical approach to determining costs and frequency of adverse drug events in a health care network. Am J Health Syst Pharm. 2001;58(12):1126–32. 10.1093/ajhp/58.12.1126 11449856

[64] McDonnell PJ , Jacobs MR . Hospital admissions resulting from preventable adverse drug reactions. Ann Pharmacother. 2002;36(9):1331–6. 10.1345/aph.1A333 12196047

[65] Cutler DM , Everett W . Thinking outside the pillbox — medication adherence as a priority for health care reform. N Engl J Med. 2010;362(17):1553–55. 10.1056/NEJMp1002305 20375400

[66] Kane JM , Kishimoto T , Correll CU . Non‐adherence to medication in patients with psychotic disorders: epidemiology, contributing factors and management strategies. World Psychiatry. 2013;12(3):216–26. 10.1002/wps.20060 24096780 PMC3799245

[67] Tiihonen J , Haukka J , Taylor M , Haddad PM , Patel MX , Korhonen P . A nationwide cohort study of oral and depot antipsychotics after first hospitalization for schizophrenia. Am J Psychiatry. 2011;168(6):603–9. 10.1176/appi.ajp.2011.10081224 21362741

[68] Rubio JM , Taipale H , Tanskanen A , Correll CU , Kane JM , Tiihonen J . Long‐term continuity of antipsychotic treatment for schizophrenia: a nationwide study. Schizophr Bull. 2021;47(6):1611–20. 10.1093/schbul/sbab063 34129663 PMC8530382

[69] Schoeler T , Petros N , Di Forti M , Klamerus E , Foglia E , Murray R , et al. Poor medication adherence and risk of relapse associated with continued cannabis use in patients with first‐episode psychosis: a prospective analysis. The Lancet Psychiatry. 2017;4(8):627–33. 10.1016/S2215-0366(17)30233-X 28705600 PMC5522816

[70] Lopez LV , Shaikh A , Merson J , Greenberg J , Suckow RF , Kane JM . Accuracy of clinician assessments of medication status in the emergency setting: a comparison of clinician assessment of antipsychotic usage and plasma level determination. J Clin Psychopharmacol. 2017;37(3):310–4. 10.1097/JCP.0000000000000697 28353490

[71] Kane JM , Schooler NR , Marcy P , Achtyes ED , Correll CU , Robinson DG . Patients with early‐phase schizophrenia will accept treatment with sustained‐release medication (long‐acting injectable antipsychotics): results from the recruitment phase of the PRELAPSE trial. J Clin Psychiatry. 2019;80(3):8m12546. 10.4088/JCP.18m12546 31050233

[72] Peters‐Strickland T , Pestreich L , Hatch A , Rohatagi S , Baker RA , Docherty JP , et al. Usability of a novel digital medicine system in adults with schizophrenia treated with sensor‐embedded tablets of aripiprazole. NDT. 2016;12:2587–94. 10.2147/NDT.S116029 PMC5067053 27785036

[73] Márquez Contreras E , Márquez Rivero S , Rodríguez García E , López‐García‐Ramos L , Carlos Pastoriza Vilas J , Baldonedo Suárez A , et al. Specific hypertension smartphone application to improve medication adherence in hypertension: a cluster‐randomized trial. Curr Med Res Opin. 2019;35(1):167–73. 10.1080/03007995.2018.1549026 30431384

[74] Tiihonen J , Mittendorfer‐Rutz E , Majak M , Mehtälä J , Hoti F , Jedenius E , et al. Real‐ world effectiveness of antipsychotic treatments in a nationwide cohort of 29 823 patients with schizophrenia. JAMA Psychiatry. 2017;74(7):686–93. 10.1001/jamapsychiatry.2017.1322 28593216 PMC5710250

[75] Kishimoto T , Hagi K , Kurokawa S , Kane JM , Correll CU . Long‐acting injectable versus oral antipsychotics for the maintenance treatment of schizophrenia: a systematic review and comparative meta‐analysis of randomised, cohort, and pre–post studies. The Lancet Psychiatry. 2021;8(5):387–404. 10.1016/S2215-0366(21)00039-0 33862018

[76] Kishimoto T , Nitta M , Borenstein M , Kane JM , Correll CU . Long‐acting injectable versus oral antipsychotics in schizophrenia: a systematic review and meta‐analysis of mirror‐image studies. J Clin Psychiatry. 2013;74(10):957–65. 10.4088/JCP.13r08440 24229745

[77] Schoretsanitis G , Kane JM , Correll CU , Rubio JM . Predictors of lack of relapse after random discontinuation of oral and long‐acting injectable antipsychotics in clinically stabilized patients with schizophrenia: a re‐analysis of individual participant data. Schizophr Bull. Published online August 6 2021;48(2):296–306. sbab091. 10.1093/schbul/sbab091 PMC8886604 34355232

[78] Misawa F , Kishimoto T , Hagi K , Kane JM , Correll CU . Safety and tolerability of long‐acting injectable versus oral antipsychotics: a meta‐analysis of randomized controlled studies comparing the same antipsychotics. Schizophr Res. 2016;176(2–3):220–30. 10.1016/j.schres.2016.07.018 27499361

[79] Guinart D , Misawa F , Rubio JM , Pereira J , Sharma H , Schoretsanitis G , et al. Outcomes of neuroleptic malignant syndrome with depot versus oral antipsychotics: a systematic review and pooled, patient‐level analysis of 662 case reports. J Clin Psychiatry. 2020;82(1):20r13272. 10.4088/JCP.20r13272 33238083

[80] Guinart D , Taipale H , Rubio JM , Tanskanen A , Correll CU , Tiihonen J , et al. Risk factors, incidence, and outcomes of neuroleptic malignant syndrome on long‐acting injectable vs oral antipsychotics in a nationwide schizophrenia cohort. Schizophr Bull. 2021;47(6):1621–30. 10.1093/schbul/sbab062 34013325 PMC8530388

[81] Buchanan RW , Kreyenbuhl J , Kelly DL , Noel JM , Boggs DL , Fischer BA , et al. The 2009 schizophrenia PORT psychopharmacological treatment recommendations and summary statements. Schizophr Bull. 2010;36(1):71–93. 10.1093/schbul/sbp116 19955390 PMC2800144

[82] Kane JM , Schooler NR , Marcy P , Correll CU , Achtyes ED , Gibbons RD , et al. Effect of long‐acting injectable antipsychotics vs usual care on time to first hospitalization in early‐phase schizophrenia: a randomized clinical trial. JAMA Psychiatry. Published online July 15 2020;77(12):1217–24. 10.1001/jamapsychiatry.2020.2076 32667636 PMC7364341

[83] Rubio JM , Kane JM . Psychosis breakthrough on antipsychotic maintenance medication (BAMM): what can we learn? NPJ Schizophr. 2017;3(1):36. 10.1038/s41537-017-0039-z 29021565 PMC5636818

[84] Rubio JM , Schoretsanitis G , John M , Tiihonen J , Taipale H , Guinart D , et al. Psychosis relapse during treatment with long‐acting injectable antipsychotics in individuals with schizophrenia‐spectrum disorders: an individual participant data meta‐analysis. The Lancet Psychiatry. 2020;7(9):749–61. 10.1016/S2215-0366(20)30264-9 32828165

[85] Rubio JM , Taipale H , Correll CU , Tanskanen A , Kane JM , Tiihonen J . Psychosis breakthrough on antipsychotic maintenance: results from a nationwide study. Psychol Med. 2020;50(8):1356–67. 10.1017/S0033291719001296 31190660

[86] Højlund M , Kemp AF , Haddad PM , Neill JC , Correll CU . Standard versus reduced dose of antipsychotics for relapse prevention in multi‐episode schizophrenia: a systematic review and meta‐analysis of randomised controlled trials. The Lancet Psychiatry. 2021;8(6):471–86. 10.1016/S2215-0366(21)00078-X 34023019

[87] Leucht S , Bauer S , Siafis S , Hamza T , Wu H , Schneider‐Thoma J , et al. Examination of dosing of antipsychotic drugs for relapse prevention in patients with stable schizophrenia: a meta‐analysis. JAMA Psychiatry. 2021;78(11):1238–48. 10.1001/jamapsychiatry.2021.2130 34406325 PMC8374744

[88] Leucht S , Tardy M , Komossa K , Heres S , Kissling W , Salanti G , et al. Antipsychotic drugs versus placebo for relapse prevention in schizophrenia: a systematic review and meta‐analysis. Lancet. 2012;379(9831):2063–71. 10.1016/S0140-6736(12)60239-6 22560607

[89] Harrow M , Jobe TH , Faull RN , Yang J . A 20‐year multi‐followup longitudinal study assessing whether antipsychotic medications contribute to work functioning in schizophrenia. Psychiatry Res. 2017;256:267–74. 10.1016/j.psychres.2017.06.069 28651219 PMC5661946

[90] Wunderink L , Nieboer RM , Wiersma D , Sytema S , Nienhuis FJ . Recovery in remitted first‐episode psychosis at 7 years of follow‐up of an early dose reduction/discontinuation or maintenance treatment strategy: long‐term follow‐up of a 2‐year randomized clinical trial. JAMA Psychiatry. 2013;70(9):913–20. 10.1001/jamapsychiatry.2013.19 23824214

[91] Moncrieff J . Antipsychotic maintenance treatment: time to rethink? PLoS Med. 2015;12(8):e1001861. 10.1371/journal.pmed.1001861 26241954 PMC4524699

[92] Moncrieff J . Does antipsychotic withdrawal provoke psychosis? Review of the literature on rapid onset psychosis (supersensitivity psychosis) and withdrawal‐related relapse. Acta Psychiatr Scand. 2006;114(1):3–13. 10.1111/j.1600-0447.2006.00787.x 16774655

[93] Kim S , Shin SH , Santangelo B , Veronese M , Kang SK , Lee JS , et al. Dopamine dysregulation in psychotic relapse after antipsychotic discontinuation: an [18F]DOPA and [11C]raclopride PET study in first‐episode psychosis. Mol Psychiatry. 2020;26:3476–88. 10.1038/s41380-020-00879-0 32929214

[94] Rubio J , Correll CU* , Kane JM . What is the risk‐benefit ratio of long‐term antipsychotic treatment in people with schizophrenia? World Psychiatry. 2018;17(2):149–60. 10.1002/wps.20516 29856543 PMC5980517

[95] Goff DC , Falkai P , Fleischhacker WW , Girgis RR , Kahn RM , Uchida H , et al. The long‐term effects of antipsychotic medication on clinical course in schizophrenia. Am J Psychiatry. Published online May 5 2017;174:840–9. appiajp201716091016. 10.1176/appi.ajp.2017.16091016 28472900

[96] Wunderink L , Nienhuis FJ , Sytema S , Slooff CJ , Knegtering R , Wiersma D . Guided discontinuation versus maintenance treatment in remitted first‐episode psychosis: relapse rates and functional outcome. J Clin Psychiatry. 2007;68(5):654–61. 17503973 10.4088/jcp.v68n0502

[97] Bowtell M , Eaton S , Thien K , Bardell‐Williams M , Downey L , Ratheesh A , et al. Rates and predictors of relapse following discontinuation of antipsychotic medication after a first episode of psychosis. Schizophr Res. 2018;195:231–6. 10.1016/j.schres.2017.10.030 29066258

[98] Insel TR , Scolnick EM . Cure therapeutics and strategic prevention: raising the bar for mental health research. Mol Psychiatry. 2006;11(1):11–7. 10.1038/sj.mp.4001777 16355250 PMC1586099

[99] Tandon R . Antipsychotics in the treatment of schizophrenia: an overview. J Clin Psychiatry. 2011;72(Suppl 1):4–8. 10.4088/JCP.10075su1.01 22217436

[100] Keltner NL , Johnson V . Biological perspectives. Perspect Psychiatr Care. 2002;38(4):157–9. 10.1111/j.1744-6163.2002.tb01566.x 12629954

[101] Brannan SK , Sawchak S , Miller AC , Lieberman JA , Paul SM , Breier A . Muscarinic cholinergic receptor agonist and peripheral antagonist for schizophrenia. N Engl J Med. 2021;384(8):717–26. 10.1056/NEJMoa2017015 33626254 PMC7610870

[102] Koblan KS , Kent J , Hopkins SC , Krystal JH , Cheng H , Goldman R , et al. A non‐D2‐ receptor‐binding drug for the treatment of schizophrenia. N Engl J Med. 2020;382(16):1497–506. 10.1056/NEJMoa1911772 32294346

[103] Forrer GR . Atropine toxicity in the treatment of mental disease. AJP. 1951;108(2):107–12. 10.1176/ajp.108.2.107 14857212

[104] Crook JM , Tomaskovic‐Crook E , Copolov DL , Dean B . Low muscarinic receptor binding in prefrontal cortex from subjects with schizophrenia: a study of Brodmann's areas 8, 9, 10, and 46 and the effects of neuroleptic drug treatment. AJP. 2001;158(6):918–25. 10.1176/appi.ajp.158.6.918 11384900

[105] Crook JM , Tomaskovic‐Crook E , Copolov DL , Dean B . Decreased muscarinic receptor binding in subjects with schizophrenia: a study of the human hippocampal formation. Biol Psychiatry. 2000;48(5):381–8. 10.1016/s0006-3223(00)00918-5 10978721

[106] Clinicaltrials.gov. https://clinicaltrials.gov/ct2/results?cond=%26term=xanomeline%26cntry=%26state=%26city=%26dist=. Accessed November 29 2021.

[107] Clinicaltrials.gov. https://clinicaltrials.gov/ct2/results?cond=%26term=SEP-363856%26cntry=%26state=%26city=%26dist=. Accessed November 29 2021.

[108] Galling B , Vernon JA , Pagsberg AK , Wadhwa A , Grudnikoff E , Seidman AJ , et al. Efficacy and safety of antidepressant augmentation of continued antipsychotic treatment in patients with schizophrenia. Acta Psychiatr Scand. 2018;137(3):187–205. 10.1111/acps.12854 29431197

[109] Solmi M , Fornaro M , Toyoshima K , Carvalho AF , Köhler CA , Veronese N , et al. Systematic review and exploratory meta‐analysis of the efficacy, safety, and biological effects of psychostimulants and atomoxetine in patients with schizophrenia or schizoaffective disorder. CNS Spectr. 2019;24(5):479–95. 10.1017/S1092852918001050 30460884

[110] Petrides G , Malur C , Braga RJ , Bailine SH , Schooler NR , Malhotra AK , et al. Electroconvulsive therapy augmentation in clozapine‐resistant schizophrenia: a prospective, randomized study. Am J Psychiatry. 2015;172(1):52–8. 10.1176/appi.ajp.2014.13060787 25157964

[111] Chang JS , Ahn YM , Park HJ , Lee KY , Kim SH , Kang UG , et al. Aripiprazole augmentation in clozapine‐treated patients with refractory schizophrenia: an 8‐week, randomized, double‐blind, placebo‐controlled trial. J Clin Psychiatry. 2008;69(5):720–31. 18370574 10.4088/jcp.v69n0505

